# Adjustments in Torque Steadiness During Fatiguing Contractions Are Inversely Correlated With IQ in Persons With Multiple Sclerosis

**DOI:** 10.3389/fphys.2018.01404

**Published:** 2018-10-17

**Authors:** Jeffrey R. Gould, Andrew E. Reineberg, Brice T. Cleland, Kristi E. Knoblauch, Grace K. Clinton, Marie T. Banich, John R. Corboy, Roger M. Enoka

**Affiliations:** ^1^Department of Integrative Physiology, University of Colorado Boulder, Boulder, CO, United States; ^2^Department of Psychology and Neuroscience, University of Colorado Boulder, Boulder, CO, United States; ^3^Department of Neurology, Anschutz Medical Campus, University of Colorado Denver, Boulder, CO, United States

**Keywords:** multiple sclerosis, fatigue, performance fatigability, perceived exertion, force steadiness, EMG, cognitive reserve

## Abstract

Fatigue is one of the most debilitating symptoms of multiple sclerosis (MS), and the underlying mechanisms are poorly understood. When exposed to a physical or cognitive challenge, individuals with MS tend to exhibit greater declines in task performance (performance fatigability) and increased levels of self-reported fatigue (perceived fatigability), but these effects may be attenuated by greater intellectual capacity. The purpose of our study was to examine the influence of intelligence on fatigability in persons with MS. We hypothesized that greater intellectual capacity confers some protection against heightened levels of fatigue and fatigability associated with MS. Twelve adults with relapsing-remitting MS were compared with 12 control (CO) subjects who were matched for age, sex, and premorbid intellectual capacity. Performance fatigability was measured as the decline in maximal voluntary contraction (MVC) torque after 60 isometric contractions (10 s contraction at 25% MVC, 5 s rest) performed with the knee extensor muscles. Perceived fatigability was assessed with the modified fatigue impact scale (MFIS) questionnaire (trait fatigability) and the Borg rating of perceived exertion (RPE, state fatigability). Persons with MS reported greater MFIS scores (MS: 43 ± 14; CO: 11 ± 8, *P* ≤ 0.001). Initial MVC torque for the knee extensors did not differ between the groups (MS: 112 ± 38 N⋅m; CO: 107 ± 44 N⋅m) and the decline (performance fatigability) was similar for both groups (MS: -16 ± 19 N⋅m; CO: -13 ± 16 N⋅m). RPE increased during the fatiguing contraction for both groups (*P* < 0.001) but was significantly greater in magnitude (main effect for group, *P* = 0.03) and increased more for the MS group (group × time interaction, *P* = 0.05). Torque steadiness declined during the fatiguing contractions (main effect for time, *P* = 0.05) and was less steady for the MS group (main effect for group, *P* = 0.02). Performance and full-4 IQ was correlated with the decline in torque steadiness for the MS group (*r* = -0.63, *P* < 0.05; *r* = -0.64, *P* < 0.05). Intellectual capacity was not associated with fatigability in persons with MS but was associated with adjustments in muscle activation during the fatiguing contractions.

## Introduction

Multiple sclerosis (MS) is a neurological disorder that compromises the integrity of signaling pathways in the central nervous system through chronic inflammatory responses and neurodegeneration (Trapp and Nave, 2008). Although the clinical progression of MS varies widely between individuals (Lublin and Reingold, 1996; Confavreux and Vukusic, 2006), one of the most common symptoms of the disease is a heightened level of fatigue (Fisk et al., 1994; Alvarenga-Filbo et al., 2015; Loy et al., 2017; Penner and Paul, 2017), the underlying mechanisms of which are poorly understood.

The word *fatigue* is commonly used throughout the literature to describe a wide variety of conditions, which leads to uncertainty in its intended meaning when it is not clearly defined. In an effort to remain consistent with more recent suggestions to adopt a unified taxonomy (Kluger et al., 2013; Enoka and Duchateau, 2016; Zijdewind et al., 2016), we define fatigue as a symptom that emerges in an individual due to adaptations in both perceived fatigability and performance fatigability. Perceived fatigability is assessed by self-report and reflects challenges to homeostasis and the psychological state of the individual. Trait levels of perceived fatigability refer to sensations reported by an individual over longer durations of time (days to weeks), whereas state levels indicate a momentary value. Performance fatigability is quantified as a decline in the ability to perform a given task due to adjustments in muscle activation and contractile capacity.

Symptoms of fatigue can severely limit activities of daily life and are often reported to have a more negative impact on quality of life than physical disability (Fisk et al., 1994; Amato et al., 2001; Janardhan and Bakshi, 2002; Alvarenga-Filbo et al., 2015). In addition to the debilitating effects of fatigue, MS often leads to various other cognitive and motor impairments. Cognitive impairments may include declines in processing speed, memory and executive function (Chiaravalloti and DeLuca, 2008), and motor impairments commonly include declines in mobility, strength, force steadiness, and performance fatigability (Koch et al., 2007; Dalgas et al., 2008; Hadjimichael et al., 2008). Some studies, however, have shown that possessing a higher premorbid intellectual capacity may confer protection against declines in cognitive function with progression of a neurological disease, an idea referred to as cognitive reserve. The concept of cognitive reserve posits that cognitive impairments develop more gradually as a neurological disease progresses in people with greater intellectual capacity (Stern, 2009). Persons with MS who have greater intellectual capacity, for example, can achieve comparable levels of performance on behavioral tasks with less modulation of cortical activity compared with those who have fewer years of education or lower vocabulary scores (Benedict et al., 2010; Sumowski et al., 2010).

Although current evidence on the relation between perceived fatigability and cognitive function appears equivocal (Morrow et al., 2009), persons with MS often report that fatigue interferes with cognitive functioning (Krupp et al., 1988; Monks, 1989) and that increases in fatigue occur after experiencing a cognitive challenge (Bailey et al., 2007; Tartaglia et al., 2008). Furthermore, a cognitive challenge worsens motor performance in individuals with MS more so than control subjects, and the increased cognitive demand is associated with elevated levels of perceived fatigability (Wolkorte et al., 2015). These findings suggest that intellectual capacity may be related to perceived and performance fatigability.

The purpose of our study was to examine the influence of intellectual capacity on perceived and performance fatigability in persons with MS. Intellectual capacity was measured by the Wechsler Abbreviated Scale of Intelligence. Given that intellectual capacity protects against MS-related declines in cognitive function (Sumowski et al., 2009a,b) and cerebral efficiency (Sumowski et al., 2010) and that fatigue is associated with greater levels of cortical activity (Enoka and Duchateau, 2016; Zijdewind et al., 2016; Loy et al., 2017; Severijns et al., 2017), we hypothesized that individuals with MS who had higher levels of intellectual capacity would experience lower levels of fatigue and fatigability.

## Materials and Methods

Twelve adults (39 ± 15 years, 1 man) with a diagnosis of relapsing-remitting MS and 12 control subjects (41 ± 14 years, 1 man) who were matched for age, sex, and intellectual capacity participated in the study after written informed consent was obtained. Participants with MS were included if they did not have a change in prescription medications (for ≥90 days), were able to walk ≥100 m unassisted, complained that fatigue interfered with their quality of life, and did not experience a relapsing episode within 90 days of participating in the study. None of the participants had a history of cardiovascular incidents, seizures, or traumatic brain injuries, and all participants were right-handed.

The experimental protocol was approved by the Institutional Review Board at the University of Colorado Boulder (protocol#: 12-0421) and conformed to the *Declaration of Helsinki*. The study involved one visit to the laboratory, which lasted ∼2 h. Intellectual capacity, executive function, walking performance, perceived fatigability, and performance fatigability were assessed during this visit.

### Intellectual Capacity

Intellectual capacity was assessed with the Wechsler Abbreviated Scale of Intelligence (WASI) (Wechsler, 1999). The test provides a performance intelligence quotient (IQ) score derived from the following: the block design and matrix reasoning subtests, a verbal IQ score based on the vocabulary and similarities subtests, and a Full-4 scale that is a composite of the performance and verbal IQ scores. Verbal performance is resistant to declines caused by the progression of MS and was therefore used to estimate premorbid intellectual capacity (Lezak, 2004; Chiaravalloti and DeLuca, 2008; Sumowski et al., 2009a) and to match control participants with those in the MS group. The Behavior Rating Inventory of Executive Function (BRIEF) served as an additional measure of cognitive function by assessing difficulties in goal-directed behavior during activities of daily living (Roth et al., 2005).

### Walking Performance

The 25-foot walk test was used to measure functional capacity of the lower extremities and was performed without the use of orthotic or other assistive devices. Participants were directed to one end of a 25-foot walkway, which was clearly marked with white lines at both ends. The instructions were to walk as quickly as possible, but safely, from one end to the other, and without slowing down until well past the end line. Measurements were made by one of the investigators using a digital stopwatch. The walk was performed a second time in the opposite direction, and the faster of the two times is reported.

### Perceived Fatigability

Trait levels of fatigue experienced by participants were quantified using the Modified Fatigue Impact Scale (MFIS) questionnaire, which considers physical, cognitive, and social factors influencing perceived fatigability (Kos et al., 2006). Participants were asked to indicate how often fatigue has had an influence on 21 different situations over the 4 weeks immediately preceding the study. Additionally, participants were asked to report the state level of fatigue at seven instances during a series of fatiguing contractions using the modified Borg scale (6–20) (Borg et al., 1987).

### Performance Fatigability

Participants performed 60 intermittent isometric contractions with the knee extensor muscles. After determining maximal voluntary contraction (MVC) torque, participants were asked to increase knee extensor torque to the target (25% MVC torque) displayed on the monitor (gain: 3% MVC force/cm) in front of them and to hold it as steady as possible for 10 s. The investigator verbally prompted the participant to begin each contraction and then to relax for 5 s. This procedure was repeated for 60 contractions. The protocol was performed with both the left and right legs in a randomized order with ∼ 20 min of rest between the two trials. MVC torque was measured before and after the 60 contractions. Performance fatigability was quantified as the change in MVC torque from before to after the intermittent isometric contractions. Rating of perceived exertion (RPE) was measured with the modified Borg scale (6–20) after every 10th contraction. The scale was anchored with six representing rest or no exertion, and 20 corresponding to the strongest possible effort.

### Experimental Setup

Participants were seated in an upright position with hips and knees flexed to ∼1.57 rad. A strain gauge force transducer (JR3, Woodland, CA, United States) was positioned to contact the anterior aspect of the lower leg (∼8 cm above the ankle). The applied force was displayed on a monitor located at eye level ∼60 cm in front of the subject. The distance between the axis of rotation for the knee joint and the horizontal plane of the lower leg that contacted the center of the force transducer was measured and used to calculate torque about the knee joint. The upper body was secured to the chair using 5 cm wide nylon straps across the shoulders and lap in order to minimize movement during contractions.

Electromyographic (EMG) signals were recorded from the vastus lateralis muscle using Ag-AgCl surface electrodes (Covidien, Mansfield, MA, United States) arranged in a bipolar configuration. Three pairs of electrodes were placed over the vastus lateralis along a line between the anterior superior iliac spine and the lateral border of the patella in the presumed direction of the muscle fibers. Three reference electrodes were placed over the medial surface of the tibia. EMG signals were amplified (1000×), band-pass filtered (13–1000 Hz) (Coulbourn Instruments, Allentown, PA, United States), sampled at 2 kHz (Cambridge Electronic Design, Cambridge, England, United Kingdom), and stored on a computer (Dell, Plano, TX, United States).

### Maximal Voluntary Contractions

Maximal voluntary contraction torque was measured before the 60 contractions to determine maximal knee extensor strength, to provide a reference value for calculating a target torque of 25% MVC, and to record peak EMG activity. MVC torque was measured again immediately after the 60 contractions to assess performance fatigability. MVCs were performed with the knee extensor muscles by gradually increasing torque from rest to maximum over ∼3 s and maintaining this torque for ∼3 s. The investigators provided strong verbal encouragement during each MVC, and the gain of the visual feedback was adjusted between trials. At least two trials were performed with ≥90 s of rest between consecutive trials. If peak torques were not within 5% of each other for the two trials, or if a participant indicated that one of the efforts was not maximal, additional MVCs were performed until these criteria were met. The greatest peak torque during these trials was used as the maximal value.

### Data Analysis

Force and EMG data were digitized (Power 1401; Cambridge Electronic Design, Cambridge, England, United Kingdom) and stored on a computer for offline analysis using Spike2 data acquisition/analysis software (Cambridge Electronic Design, Cambridge, England, United Kingdom). Force signals were sampled at 1 kHz.

The maximal EMG amplitude during the MVC trial with the maximal torque was quantified as the root-mean-square (RMS) value during a 0.5 s interval that spanned the peak of the EMG. This value was used to normalize subsequent EMG recordings. EMG data recorded during the 60 contractions were quantified as the normalized RMS value during 8 s intervals for the first, 10th, 20th, 30th, 40th, 50th, and 60th contraction. Torque steadiness was quantified during the same 8-s intervals as the coefficient of variation for torque [% = (SD/mean) × 100; Galganski et al. (1993)].

### Statistical Analysis

Dependent *t*-tests were used to compare dependent variables before and after the 60 contractions and independent *t*-tests were used to compare group differences of dependent variables and subject characteristics. Two-factor analysis of variance was used to examine changes in dependent variables measured across the 60 contractions (first contraction, and every tenth contraction up to the 60th contraction) and differences between the two groups (MS and Control). Dependent variables measured during the fatigue protocol included EMG amplitude for the vastus lateralis muscle, knee extensor torque steadiness (the coefficient of variation for torque), and ratings of perceived exertion. One of the subjects in the MS group was unable to complete all 60 contractions, so data obtained during the last performed contraction were also used for the 50th and 60th contractions. Data collected on both legs were averaged for each participant as there were no significant differences between the left and right legs. Spearman’s correlation coefficients were determined between all measures of physical and cognitive function. Correlations were performed separately for the two groups. A significance level for all statistical tests was set at *P* ≤ 0.05. Data are presented as mean ± standard deviation (SD) in the text and as mean ± standard error (SE) in the figures.

## Results

**FIGURE 1:**
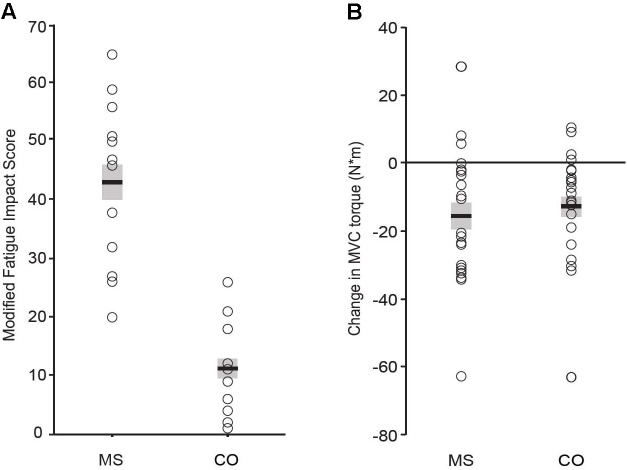
Perceived trait fatigue **(A)**. Reported levels of fatigue (MFIS score) were greater for the MS group (*P* = 0.001). *Performance fatigability* **(B)**. Declines in MVC torque were significant (*P* = 0.001 for both groups) and did not differ between groups (*P* = 0.74).

By design, individuals with MS reported significantly greater levels of trait fatigue than the control (CO) group, as measured with the MFIS questionnaire (**Figure 1A**; MS: 43 ± 14; CO: 11 ± 8, *P* ≤ 0.001). In contrast, there were no statistically significant differences between MS and CO groups for verbal IQ (**Table 1**; MS: 112 ± 13; CO: 114 ± 10), as the two groups were matched for this attribute. Performance IQ (MS: 119 ± 14; CO: 122 ± 11) and Full-4 IQ (MS: 118 ± 14; CO: 120 ± 10) also did not differ significantly between the two groups. The overall intellectual capacity of the MS group, therefore, was similar to that for the CO group, despite the MS participants having been diagnosed with the disease for 9.4 ± 6.4 years (range: 0.2–19 years) prior to participating in the study. However, a measure of executive function (BRIEF) was different between groups (MS: 122 ± 19; CO: 90 ± 17, *P* ≤ 0.001), indicating lower executive function in the MS participants. Greater BRIEF scores were correlated with greater levels of trait fatigue (MFIS), but this relation was only significant (*P* < 0.05) for the CO group (**Table 2**).

**Table 1:** Subject characteristics.

	Multiple sclerosis (MS) *n =* 12, 1 man	Control (CO) *n =* 12, 1 man
Age (years)	39 ± 15	41 ± 14
Height (cm)	164 ± 5	169 ± 5
Body mass (kg)	58 ± 4	61 ± 8
MFIS	43 ± 14	11 ± 8^†^
WASI-Verbal	112 ± 13	114 ± 10
WASI-Performance	120 ± 14	122 ± 11
WASI-Full-4	118 ± 14	120 ± 10
BRIEF	122 ± 19	90 ± 17^†^
25-foot walk (s)	3.6 ± 0.4	3.3 ± 0.4
MVC torque (N⋅m)	112 ± 38	107 ± 44
MVC decline (N⋅m)	16 ± 19	13 ± 16


*MFIS, Modified Fatigue Impact Scale; WASI, Wechsler Abbreviated Scale of Intelligence; BRIEF, Behavior Rating Inventory of Executive Function; MVC torque indicates the strength of the knee extensor muscle before the 60 isometric contractions, whereas MVC decline refers to the observed decrease in torque after the contractions. ^†^P ≤ 0.001 between MS and CO groups.*

**Table 2:** Correlation coefficients for pairs of variables are shown for both multiple sclerosis (MS) subjects and control (CO) subjects.

	Verbal IQ	Performance IQ	Full-4 IQ	BRIEF	ΔRPE	Δ MVC	Δ EMG	Δ CV for torque
MFIS	0.06/0.09	–0.36/0.12	–0.15/–0.05	0.33/0.60^∗^	–0.27/–0.10	–0.22/0.45	0.24/0.41	0.10/0.19
Verbal IQ		0.74^†^/0.55	0.88^‡^/0.86^†^	–0.23/–0.09	–0.25/–0.12	0.04/0.49	0.11/–0.13	–0.49/0.02
Performance IQ			0.94^‡^/0.80^†^	–0.48/–0.20	–0.21/–0.27	0.15/0.06	–0.24/–0.31	–0.63^∗^/–0.03
Full-4 IQ				–0.36/–0.14	–0.24/–0.19	0.13/0.34	–0.17/–0.11	–0.64^∗^/0.12
BRIEF					0.30/0.43	0.17/0.41	0.34/0.46	0.57/0.57
Δ RPE						0.21/0.23	0.50/–0.01	0.56/0.20
Δ MVC							0.34/0.45	0.23/0.50
Δ EMG								0.73^†^/0.61^∗^


*Spearman’s rank correlations for each pair of variables are presented in the table as MS/CO. Changes in rating of perceived exertion (RPE), maximal voluntary contraction (MVC) torque, electromyography for vastus lateralis (EMG), and coefficient of variation for torque (CV for Torque) refer to differences (Δ) between initial and final measurements. ^∗^P ≤ 0.05, ^†^P ≤ 0.01 and ^‡^P ≤ 0.001.*

There were no statistically significant group differences in either the 25-foot walk time (MS: 3.6 ± 0.4 s; CO: 3.3 ± 0.4 s) or knee extensor MVC torque prior to the 60 contractions (MS: 112 ± 38 N⋅m; 107 ± 44 N⋅m). Participants performed the 60 contractions with both legs separately due to the possibility of disease progression having an unequal effect on both sides of the body (Chung et al., 2008). However, changes in EMG, RPE, coefficient of variation for torque, and declines in MVC torque did not differ between left and right legs for either group of participants, so data for both legs were averaged for each participant. The two groups exhibited similar significant declines in MVC torque after the 60 contractions (**Figure 1B**; MS: -16 ± 19 N⋅m; CO: -13 ± 16 N⋅m).

**FIGURE 2:**
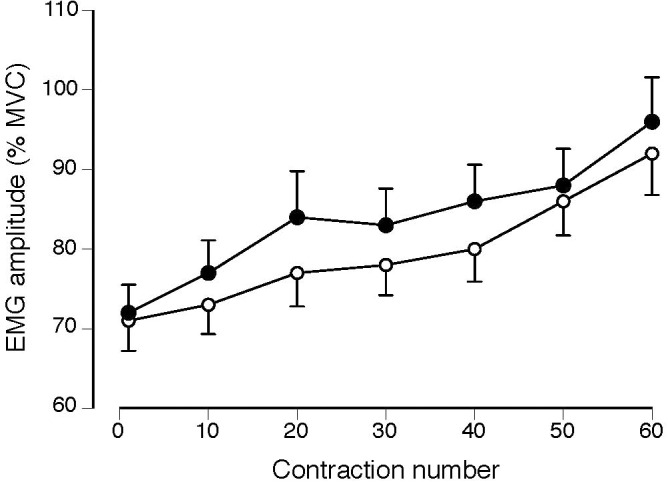
Normalized EMG amplitude for the vastus lateralis muscle in MS (filled circles) and CO subjects (open circles) increased during the 60 contractions (*P* < 0.001) with no difference between groups (*P* = 0.49).

**FIGURE 3:**
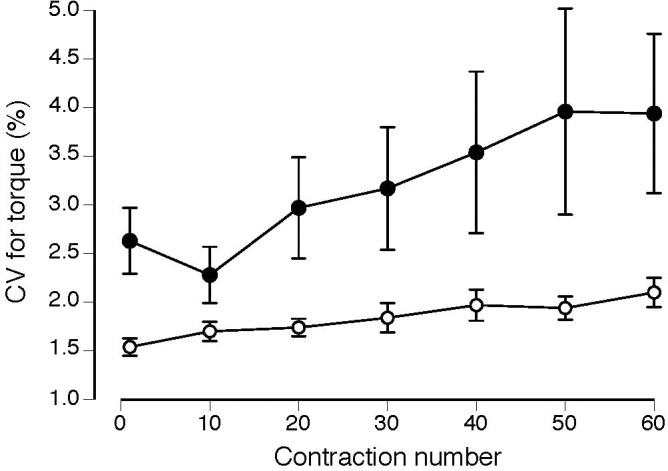
Coefficient of variation (CV) for torque increased during the 60 contractions (main effect for time, *P* = 0.05) for the MS (filled circles) and CO subjects (open circles) and the MS group was less steady (greater CV for torque) throughout the task (main effect for group, *P* = 0.02).

Normalized EMG amplitude for vastus lateralis (MS: 38 ± 37% increase; CO: 31 ± 24% increase) increased, similarly, between groups (*P* = 0.49) during the 60 contractions (**Figure 2**; *P* < 0.001). Although the coefficient of variation for torque increased for both groups (MS: 66 ± 187% increase; CO: 40 ± 49% increase) during the 60 contractions (**Figure 3**; main effect for time, *P* = 0.05), the MS group was less steady (main effect for group, *P* = 0.02). Increases in coefficient of variation for torque were strongly correlated with increases in EMG amplitude for both MS (*r* = 0.73, *P* ≤ 0.001) and CO (*r* = 0.61, *P* ≤ 0.05) groups (**Table 2**). In addition, the increase in the coefficient of variation for torque during the 60 contractions observed for the MS group (start: 2.5 ± 1.6%; end: 3.9 ± 4.3%) was inversely correlated (**Table 2**) with two measures of intellectual capacity (Performance IQ: *r* = -0.63, *P* ≤ 0.05; Full-4 IQ: *r* = -0.64, *P* ≤ 0.05), but not verbal IQ.

**FIGURE 4:**
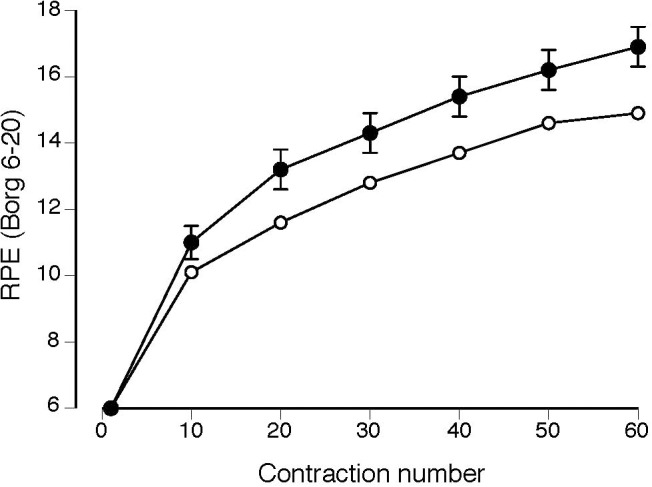
Rating of perceived exertion (RPE) increased more for the MS group (filled circles) during the 60 isometric contractions (group × time interaction, *P* = 0.05) than for the control group (open circles). Values reported as mean ± SE.

Rating of perceived exertion increased for both groups during the 60 contractions (**Figure 4**; *P* < 0.001), but was significantly greater in magnitude (group effect, *P* = 0.03) and increased more for the MS group than the CO group (group × time interaction, *P* = 0.05). The MS group tended to exhibit a greater incidence of reaching maximal effort with 5 participants reaching a final RPE of 20 at the end of the 60 contractions and only one participant for the CO group (χ^2^ = 3.6, *P* = 0.06).

## Discussion

MS participants had greater trait levels of perceived fatigability (MFIS) and greater state levels of perceived fatigability (RPE) during the intermittent contractions, but similar levels of performance fatigability (decline in MVC force) compared with the control group. Contrary to our hypothesis, there was no association between intellectual capacity and either perceived or performance fatigability. Our main finding was that torque steadiness worsened at a greater rate in the MS group during the fatiguing contractions with the knee extensors and that the change in torque steadiness for the MS group was inversely related to intellectual capacity as measured by performance IQ and full-4 IQ, but not verbal IQ. There were no group differences for Performance IQ suggesting that cognitive function, as measured by performing the block design and matrix reasoning tests, was spared in this sample of MS participants. However, differences in performance IQ within the MS group were associated with a reduced ability to maintain a steady muscle contraction with the knee extensors. Further, MS participants had greater impairments in executive function as measured by the BRIEF score. These findings indicate that intellectual capacity as measured by verbal and performance IQ appear to be retained despite 9.4 ± 6.4 years (range: 0.2–19 years) of disease progression, however, executive function may be impaired earlier by MS.

### Muscle Activation and Intelligence

Due to the association between the increase in perceived fatigability (RPE) and EMG amplitude during submaximal fatiguing contractions (Hunter et al., 2002; Rudroff et al., 2007), the more rapid increase in RPE for the MS group in the current study should have been accompanied by a quicker increase in EMG amplitude during the fatigue protocol. In contrast, the increase in EMG amplitude for the vastus lateralis muscle was similar for the two groups (**Figure 2**). However, normalized measures of EMG amplitude as recorded with bipolar electrodes are relatively insensitive to modest changes in the underlying motor unit activity (Mottram et al., 2005; Rudroff et al., 2013; Farina et al., 2014).

Nonetheless, the more rapid increase in the coefficient of variation for torque – a measure of torque steadiness – for the MS group suggests that the neural drive to the muscles differed between the two groups during the 60 isometric contractions. The synaptic inputs received by motor neurons arise from three sources: a control signal received by all motor neurons, independent noise, and common noise (Farina and Negro, 2015; Farina et al., 2016). Although the timing of the action potentials discharged by each motor neuron is relatively independent, the control signal to produce the required muscle force (neural drive to muscle) depends on the common synaptic input received by the motor neurons. Critically, there is a strong association between the estimated variance in the common synaptic input to motor neurons and the amplitude of the force fluctuations (force steadiness) during a steady submaximal contraction (Farina et al., 2016; Feeney et al., 2018; Thompson et al., 2018). Thus, the more rapid increase in the coefficient of variation for torque during the fatigue protocol for the MS participants in the current study suggests quantitative differences between the two groups in the variance in the control signal generated by the nervous system during this task.

Consistent with the interpretation of a significant role for motor unit activity in modulating motor function in persons with MS, Almuklass et al. (2018) found that some of the variance in the performance of walking tests by individuals with MS could be explained by the discharge characteristics of motor units during steady submaximal contractions. They found that 40% of the variance in a test of walking endurance (6-min test) and maximal gait speed (25-ft test) could be explained by predictor variables that included the mean interval between consecutive action potentials (interspike interval) for soleus and medial gastrocnemius during submaximal isometric contractions, the strength of the dorsiflexor muscles, and force steadiness of the plantar flexor muscles.

Our findings indicated a strong correlation between the worsening of torque steadiness during the 60 isometric contractions and intellectual capacity for the MS group, but not the control group. Studies that have examined the interactions between cognitive and motor function have shown that the ability to exert a steady force during a voluntary contraction is compromised when the individual is exposed to a cognitive challenge (Zijdewind et al., 2006), and that this effect is greater during fatiguing contractions (Lorist et al., 2002) and in older adults (Vanden Noven et al., 2014; Pereira et al., 2015). Similarly, D’Orio et al. (2012) found that measures of processing speed, executive function, memory and general intelligence were among the cognitive variables that were significantly associated with walking speed and fall frequency in persons with MS. General intelligence as measured by the WASI test was associated with walking speed (*r* = -0.272, *P* = 0.017) as well as frequency of falls (*r* = -0.229, *P* = 0.043). Although measures of intellectual capacity were not correlated with walking performance in our study and time to complete the 25-foot walk did not differ between the two groups, intellectual capacity was inversely correlated with a worsening of torque steadiness during the 60 isometric contractions for the MS group, which may indicate that changes in the neural control of leg muscles may precede declines in walking performance.

### Fatigue and Fatigability

Despite greater trait and state levels of perceived fatigability, performance fatigability for the MS participants was not different from control participants. The groups had similar declines in knee extensor torque after the fatigue protocol and similar increases in EMG amplitude. Similarities between groups could not be explained by baseline differences in MVC torque or mobility as measured by the 25-foot walk test. Steens et al. (2012a) reported similar observations when they compared the decrease in MVC force after a 2-min sustained maximal contraction with a hand muscle exhibited by 20 persons with relapsing-remitting MS and 20 age- and sex-matched control subjects. Based on a multiple-regression analysis to explain the variance in the trait level of fatigue (Fatigue Severity Scale, FSS) for the MS participants, they found that 45% of the variance in the FSS score could be explained by the measure of performance fatigability (decline in MVC force) and normalized muscle strength (MVC force). When a measure of depression (Hospital Anxiety and Depression Scale Questionnaire) was included in the analysis, the regression model explained 77% of variance in the trait level of fatigue (FSS score).

In another study, Steens et al. (2012b) compared the adjustments exhibited by persons with relapsing-remitting MS and control subjects during the 2 min sustained maximal contraction with a hand muscle. As in their other study (Steens et al., 2012a), performance fatigability (decline in MVC force) was similar for the two groups of participants. Nonetheless, there were significant differences between the two groups in the changes in the level of voluntary activation (twitch interpolation technique) and cortical activation as determined with functional MRI. Compared with the control subjects, the MS participants exhibited a more substantial and variable reduction in voluntary activation and less of an increase in cortical activation during the fatiguing contraction. A multiple-regression analysis indicated that control subjects who were stronger experienced greater performance fatigability, as reported in other studies (Hunter and Enoka, 2001; Keller-Ross et al., 2014), and that performance fatigability for the individuals with MS was less for those who could sustain greater levels of voluntary activation (Wolkorte et al., 2016).

Based on the results of Steens et al. (2012b), a greater increase in perceived fatigability (RPE) during the fatigue protocol in our study might be explained by a tendency for persons with MS to experience a greater reduction in voluntary activation during fatiguing contractions. Thus, it is necessary for these individuals to increase the levels of cortical activation to achieve the submaximal target torque during the fatigue protocol.

### Limitations

Although our study enrolled relatively few participants with a heterogeneous disease status, one of its strengths was that the participants in the MS group reported greater levels of trait fatigue despite no significant group differences in intellectual capacity, strength, or walking speed, as such differences may have influenced measured levels of trait fatigue. Consequently, our findings may not generalize to a wider population of individuals with MS who have varying levels of disability. Also, we did not control the medications being taken by our participants to treat symptoms other than to ensure that they were on stable doses of medications.

## Conclusion

Although our study found no statistically significant associations between the trait level of fatigue and measures of performance and perceived fatigability for the MS participants during a series of intermittent isometric contractions, intellectual capacity was associated with adjustments in torque steadiness during the fatiguing contraction. This finding substantiates further inquiry into the utility of the performance IQ score to detect changes in neuromuscular function that precede physical disability due to disease progression in persons with MS.

## Author Contributions

JG contributed to the conception of the study, participant recruitment and testing, data analysis and interpretation, and writing and approval of the manuscript. AR and BC contributed to participant recruitment and testing, data analysis, and writing and approval of the manuscript. KK and GC contributed to testing, data analysis, and approval of the manuscript. MB and JC contributed to conception of the study, participant recruitment, data interpretation, and approval of the manuscript. RE contributed to conception of the study, participant recruitment, data interpretation, and writing and approval of the manuscript.

## Conflict of Interest Statement

The authors declare that the research was conducted in the absence of any commercial or financial relationships that could be construed as a potential conflict of interest.

## References

[B1] Almuklass A. M. Davis L. Hamilton L. D. Vieira T. Botter A. Enoka R. M. (2018). Motor unit discharge characteristics and walking performance of individuals with multiple sclerosis. *J. Neurophysiol.* 119 1273–1282. 10.1152/jn.00598.2017 29357453 PMC5966731

[B2] Alvarenga-Filbo H. Ponziani G. Rossi F. Liedl C. L. Stefanile C. Rossi L. (2015). Does fatigue occur in MS patients without disability? *Int. J. Neurosci.* 125 107–115. 10.3109/00207454.2014.909415 24697509

[B3] Amato M. P. Ponziani G. Rossi F. Liedl C. L. Stefanile C. Rossi L. (2001). Quality of life in multiple sclerosis: the impact of depression, fatigue and disability. *Mult. Scler.* 7 340–344. 10.1177/135245850100700511 11724451

[B4] Bailey A. Channon S. Beaumont J. G. (2007). The relationship between subjective fatigue and cognitive fatigue in advanced multiple sclerosis. *Mult. Scler.* 13 73–80. 10.1177/1352458506071162 17294614

[B5] Benedict R. H. Morrow S. A. Weinstock Guttman B. Cookfair D. Schretlen D. J. (2010). Cognitive reserve moderates decline in information processing speed in multiple sclerosis patients. *J. Int. Neuropsychol. Soc.* 16 829–835. 10.1017/S1355617710000688 20609273

[B6] Borg G. Hassmén P. Lagerström M. (1987). Perceived exertion related to heart rate and blood lactate during arm and leg exercise. *Eur. J. Appl. Physiol.* 65 679–685. 10.1007/BF00424810 3678222

[B7] Chiaravalloti N. D. DeLuca J. (2008). Cognitive impairment in multiple sclerosis. *Lancet Neurol.* 7 1139–1151. 10.1016/S1474-4422(08)70259-X 19007738

[B8] Chung L. H. Remelius J. G. Van Emmerik R. E. A. Kent-Braun J. A. (2008). Leg power asymmetry and postural control in women with multiple sclerosis. *Med. Sci. Sports Exerc.* 40 1717–1724. 10.1249/MSS.0b013e31817e32a3 18799980

[B9] Confavreux C. Vukusic S. (2006). Natural history of multiple sclerosis: a unifying concept. *Brain* 129 606–616. 10.1093/brain/awl007 16415308

[B10] Dalgas U. Stenager E. Ingemann-Hansen T. (2008). Review: multiple sclerosis and physical exercise: recommendations for the a lication of resistance-, endurance- and combined training. *Mult. Scler.* 14 35–53. 10.1177/1352458507079445 17881393

[B11] D’Orio V. L. Foley F. E. Armentano F. Picone A. M. Kim S. Holtzer R. (2012). Cognitive and motor functioning in patients with multiple sclerosis: neuropsychological predictors of walking speed and falls. *J. Neurol. Sci.* 316 42–46. 10.1016/j.jns.2012.02.003 22353853

[B12] Enoka R. M. Duchateau J. (2016). Translating fatigue to human performance. *Med. Sci. Sports Exerc.* 48 2228–2238. 10.1249/MSS.0000000000000929 27015386 PMC5035715

[B13] Farina D. Merletti R. Enoka R. M. (2014). The extraction of neural strategies from the surface EMG: an update. *J. Appl. Physiol.* 117 1215–1230. 10.1152/japplphysiol.00162.2014 25277737 PMC4254845

[B14] Farina D. Negro F. (2015). Common synaptic input to motor neurons, motor unit synchronization, and force control. *Exerc. Sport Sci. Rev.* 43 23–33. 10.1249/JES.0000000000000032 25390298

[B15] Farina D. Negro F. Muceli S. Enoka R. M. (2016). Principles of motor unit physiology evolve with advances in technology. *Physiology* 31 83–94. 10.1152/physiol.00040.2015 26889014

[B16] Feeney D. F. Mani D. Enoka R. M. (2018). Variability in common synaptic input to motor neurons modulates both force steadiness and pegboard time in young and older adults. *J. Physiol.* 596 3793–3806. 10.1113/JP275658 29882259 PMC6092304

[B17] Fisk J. D. Pontefract A. Ritvo P. G. Archibold C. J. Murray T. J. (1994). The impact of fatigue on patients with multiple sclerosis. *Can. J. Neurol. Sci.* 21 9–14. 10.1017/S0317167100048691 8180914

[B18] Galganski M. E. Fuglevand A. J. Enoka R. M. (1993). Reduced control of motor output in a human hand muscle of elderly subjects during submaximal contractions. *J. Neurophysiol.* 69 2108–2115. 10.1152/jn.1993.69.6.2108 8350134

[B19] Hadjimichael O. Vollmer J. Oleen-Burkey M. (2008). Fatigue characteristics in multiple sclerosis: the North American Research Committee on Multiple Sclerosis (NARCOMS) survey. *Health Qual. Life Outcomes* 6:100. 10.1186/1477-7525-6-100 19014588 PMC2596785

[B20] Hunter S. K. Enoka R. M. (2001). Sex differences in the fatigability of arm muscles depends on the absolute force during isometric contractions. *J. Appl. Physiol.* 91 2686–2694. 10.1152/jappl.2001.91.6.2686 11717235

[B21] Hunter S. K. Ryan D. L. Ortega J. D. Enoka R. M. (2002). Task differences with the same load torque alter the endurance time of submaximal fatiguing contractions in humans. *J. Neurophysiol.* 88 3087–3096. 10.1152/jn.00232.2002 12466432

[B22] Janardhan V. Bakshi R. (2002). Quality of life in patients with multiple sclerosis: the impact of fatigue and depression. *J. Neurol. Sci.* 205 51–58. 10.1016/S0022-510X(02)00312-X 12409184

[B23] Keller-Ross M. L. Pereira H. M. Pruse J. Yoon T. Schindler-Delap B. Nielson K. A. (2014). Stressor-induced increase in muscle fatigability of young men and women is predicted by strength but not voluntary activation. *J. Appl. Physiol.* 116 767–778. 10.1152/japplphysiol.01129.2013 24526582 PMC3972745

[B24] Kluger B. M. Krupp L. B. Enoka R. M. (2013). Fatigue and fatigability in neurologic illnesses: proposal for a unified taxonomy. *Neurology* 80 409–416. 10.1212/WNL.0b013e31827f07be 23339207 PMC3589241

[B25] Koch M. Mostert J. Heersema D. (2007). Tremor in multiple sclerosis. *J. Neurol.* 254 133–145. 10.1007/s00415-006-0296-7 17318714 PMC1915650

[B26] Kos D. Nagels G. D’Hooghe M. B. Duportail M. Kerckhofs E. (2006). A rapid screening tool for fatigue impact in multiple sclerosis. *BMC Neurol.* 6:27. 10.1186/1471-2377-6-27 16916440 PMC1579227

[B27] Krupp L. B. Alvarez L. A. LaRocca N. Scheinberg L. (1988). Fatigue in multiple sclerosis. *Arch. Neurol.* 45 435–437. 10.1001/archneur.1988.00520280085020 3355400

[B28] Lezak M. D. (2004). *Neuropsychological Assessment*, 4th Edn New York, NY: Oxford University Press.

[B29] Lorist M. M. Kernell D. Meijman T. F. Zijdewind I. (2002). Motor fatigue and cognitive task performance in humans. *J. Physiol.* 545 313–319. 10.1113/jphysiol.2002.027938 12433971 PMC2290666

[B30] Loy B. D. Taylor R. L. Fling B. W. Horak F. B. (2017). Relationship between perceived fatigue and performance fatigability in people with multiple sclerosis: a systematic review and meta-analysis. *J. Psychosom. Res.* 100 1–7. 10.1016/j.jpsychores.2017.06.017 28789787 PMC5875709

[B31] Lublin F. D. Reingold S. C. (1996). Defining the clinical course of multiple sclerosis: results of an international survey. *Neurology* 46 907–911. 10.1212/WNL.46.4.907 8780061

[B32] Monks J. (1989). Experiencing symptoms in chronic illness: fatigue in multiple sclerosis. *Int. Disabil. Study* 11 78–83. 10.3109/03790798909166394 2630556

[B33] Morrow S. A. Weinstock-Guttman B. Munschauer F. E. Hojnacki D. Benedict R. H. (2009). Subjective fatigue is not associated with cognitive impairment in multiple sclerosis: cross-sectional and longitudinal analysis. *Mult. Scler.* 15 998–1005. 10.1177/1352458509106213 19667024

[B34] Mottram C. J. Jakobi J. M. Semmler J. G. Enoka R. M. (2005). Motor-unit activity differs with load type during a fatiguing contraction. *J. Neurophysiol.* 93 1381–1392. 10.1152/jn.00837.2004 15483059

[B35] Penner I. K. Paul F. (2017). Fatigue as a symptom or comorbidity of neurological diseases. *Nat. Rev. Neurosci.* 13 662–675. 10.1038/nrneurol.2017.117 29027539

[B36] Pereira H. M. Spears V. C. Schlinder-Delap B. Yoon T. Nielson K. A. Hunter S. K. (2015). Age and sex differences in steadiness of elbow flexor muscles with imposed cognitive demand. *Eur. J. Appl. Physiol.* 115 1367–1379. 10.1007/s00421-015-3113-0 25633070 PMC4431934

[B37] Roth R. M. Isquith P. K. Gioia G. A. (2005). *Behavioral Rating Inventory of Executive Function—Adult Version.* Lutz, FL: Psychological Assessment Resources, Inc.

[B38] Rudroff T. Barry B. K. Stone A. L. Barry C. J. Enoka R. M. (2007). Accessory muscle activity contributes to the variation in time to task failure for different arm postures and loads. *J. Appl. Physiol.* 102 1000–1006. 10.1152/japplphysiol.00564.2006 17095642

[B39] Rudroff T. Kalliokoski K. K. Block D. E. Gould J. R. Klingensmith W. C. Enoka R. M. (2013). PET/CT imaging of age- and task-associated differences in muscle activity during fatiguing contractions. *J. Appl. Physiol.* 114 1211–1219. 10.1152/japplphysiol.01439.2012 23412899 PMC3656430

[B40] Severijns D. Zijdewind I. Dalgas U. Lamers I. Lismont C. Feys P. (2017). The assessment of motor fatigability in persons with multiple sclerosis: a systematic review. *Neurorehabil. Neural Repair* 31 413–431. 10.1177/1545968317690831 28413944

[B41] Steens A. de Vries A. Hemmen J. Heersema D. J. Mauritz N. Zijdewind I. (2012a). Fatigue perceived by multiple sclerosis patients is associated with muscle fatigue. *Neurorehabil. Neural Repair* 26 48–57. 10.1177/1545968311416991 21856990

[B42] Steens A. Heersema D. J. Maurits N. M. Renken R. J. Zijdewind I. (2012b). Mechanisms underlying muscle fatigue differ between multiple sclerosis patients and controls: a combined electrophysiological and neuroimaging study. *Neuroimage* 59 3110–3118. 10.1016/j.neuroimage.2011.11.038 22138253

[B43] Stern Y. (2009). Cognitive reserve. *Neuropsychologica* 47 2015–2028. 10.1016/j.neuropsychologia.2009.03.004 19467352 PMC2739591

[B44] Sumowski J. F. Chiaravalloti N. DeLuca J. (2009a). Cognitive reserve protects against cognitive dysfunction in multiple sclerosis. *J. Clin. Exp. Neuropsyc.* 31 913–926. 10.1080/13803390902740643 19330566

[B45] Sumowski J. F. Chiaravalloti N. D. Wylie G. R. DeLuca J. (2009b). Cognitive reserve moderates the negative effect of brain atrophy on cognitive efficiency in multiple sclerosis. *J. Int. Neuropsych. Soc.* 15 606–612. 10.1017/S1355617709090912 19573279

[B46] Sumowski J. F. Wylie G. R. DeLuca J. Chiaravalloti N. (2010). Intellectual enrichment is linked to cerebral efficiency in multiple sclerosis: functional magnetic resonance imaging evidence for cognitive reserve. *Brain* 133 362–374. 10.1093/brain/awp307 20008455 PMC2822636

[B47] Tartaglia M. C. Narayanan S. Arnold D. L. (2008). Mental fatigue alters the pattern and increases the volume of cerebral activation required for a motor task in multiple sclerosis patients with fatigue. *Eur. J. Neurol.* 15 413–419. 10.1111/j.1468-1331.2008.02090.x 18353127

[B48] Thompson C. K. Negro F. Johnson M. D. Holmes M. R. McPherson L. M. Powers R. K. (2018). Robust and accurate decoding of motoneuron behavior and prediction of the resulting force output. *J. Physiol.* 596 2643–2659. 10.1113/JP276153 29726002 PMC6046070

[B49] Trapp B. D. Nave K. A. (2008). Multiple sclerosis: an immune or neurodegenerative disorder? *Annu. Rev. Neurosci.* 31 247–269. 10.1146/annurev.neuro.30.051606.094313 18558855

[B50] Vanden Noven M. L. Pereira H. M. Yoon T. Stevens A. A. Nielson K. A. Hunter S. K. (2014). Motor variability during sustained contractions increases with cognitive demand in older adults. *Front. Aging Neurosci.* 6:97. 10.3389/fnagi.2014.00097 24904410 PMC4033244

[B51] Wechsler D. (1999). *Wechsler Abbreviated Scale of Intelligence.* San Antonio, TX: Psychological Corporation.

[B52] Wolkorte R. Heersema D. J. Zijdewind I. (2015). Reduced dual-task performance in MS patients is further decreased by muscle fatigue. *Neurorehabil. Neural Repair* 29 424–435. 10.1177/1545968314552529 25288582

[B53] Wolkorte R. Heersema D. J. Zijdewind I. (2016). Reduced voluntary activation during brief and sustained contractions of a hand muscle in secondary-progressive multiple sclerosis patients. *Neurorehabil. Neural Repair* 30 307–316. 10.1177/1545968315593809 26156191

[B54] Zijdewind I. Prak R. F. Wolkorte R. (2016). Fatigue and fatigability in persons with multiple sclerosis. *Exerc. Sport Sci. Rev.* 44 123–128. 10.1249/JES.0000000000000088 27465682

[B55] Zijdewind I. Van Duinen H. Zielman R. Lorist M. M. (2006). Interaction between force production and cognitive performance in humans. *Clin. Neurophysiol.* 117 660–667. 10.1016/j.clinph.2005.11.016 16434230

